# Novel Biocomposite of Starch and Flax Fiber Modified with Tannic Acid with Biocidal Properties

**DOI:** 10.3390/polym16081108

**Published:** 2024-04-16

**Authors:** Magdalena Stepczyńska, Piotr Rytlewski, Krzysztof Moraczewski, Alona Pawłowska, Tomasz Karasiewicz

**Affiliations:** Faculty of Materials Engineering, Kazimierz Wielki University, Jana Karola Chodkiewicza 30, 85-064 Bydgoszcz, Poland; prytlewski@ukw.edu.pl (P.R.); kmm@ukw.edu.pl (K.M.); alona.pawlowska@ukw.edu.pl (A.P.); tomakara@ukw.edu.pl (T.K.)

**Keywords:** thermoplastic starch, biocomposities, flax fibers, tannic acid, fibers modification, natural biocidal compounds

## Abstract

The purpose of this paper was to develop novel biocomposites with biocidal properties in microorganisms, with enhanced mechanical strength and hydrophobicity as well as with increased biodegradation rates. The main idea and the novelty of this work was to use cross-linking compounds and, at the same time, biocidal compounds—natural compounds of plant origin with biocidal properties. The authors assumed that the modification of flax fiber by natural plant compound will reduce the hydrophilicity of novel biocompositie. Biopolymer based on thermoplastic starch reinforced with flax fibres modified with tannic acid (TA) was prepared by extrusion and injection molding processes. The effects of TA modification on the mechanical and structural properties of biocomposites were analyzed through DMA, tensile tests, DSC, and TG. The biocidal and wettability properties of the biocomposites were investigated. The article also discusses the outcomes of research conducted on the structural characteristics and rates of the biodegradation of biocomposites.

## 1. Introduction

The term “biodegradable materials” commonly refers to plastics capable of rapid biodegradation under industrial composting conditions, making them promising candidates as future polymers. The production of biodegradable polymers from both natural and petrochemical resources is a topic of research in leading scientific institutions and industrial laboratories worldwide.

The rapid development of specialized applications of polymeric materials in various areas of technology and other aspects of life has increased the demand for higher quality standards. The common use of these materials has led to a larger load in the natural environment, mainly due to the growing mass of plastic waste. Therefore, there is a need to introduce mass-scale application of biodegradable materials to reduce our reliance on non-biodegradable plastic.

To meet environmental protection requirements and comply with legislation, there is a growing trend toward replacing plastics derived from crude oil with polymers made from renewable sources such as maize. These polymers decompose into minerals, water, and gases. Currently, research in the field of biodegradable materials is focused on both synthetic and natural polymers. Synthetic polymers such as polylactic acid (PLA), polycaprolactone (PCL), polyadipate butylene terephthalate (PBAT), and polyvinyl alcohol (PVA) are widely used biodegradable materials in daily life. On the other hand, natural polymers such as polysaccharides (starch, cellulose, chitosan), proteins (soy and whey protein, silk fibroin), and lipids (beeswax) are being used as raw materials for the construction of biodegradable materials [1,2].

Currently, many researchers are focusing on creating biodegradable materials using starch due to its abundance, low cost, non-toxicity, and renewability. Starch is a sustainable material derived from renewable sources such as potato, wheat, corn, rice, and tapioca [3,4,5,6]. It is not only widely used in the food industry but also in non-food applications such as packaging materials [7,8,9,10,11,12,13]. However, starch is resistant to being processed as a thermoplastic material due to intermolecular forces and hydrogen bonds. Therefore, to transform starch into thermoplastic starch (TPS), a plasticizer such as glycerin, glycerol, urea, citric acid, glucose, sorbitol, or others must be added [5,6,14]. The addition of a plasticizer enhances the processability and flexibility of TPS. However, the common use of plasticizers has a significant impact on the environment, and therefore, they should be natural, inexpensive, and renewable to make the production process cost-effective and biodegradable.

Numerous scientific and industrial projects are focused on utilizing plant-based fillers to create fully biodegradable biocomposites. Starch, wood flour, and plant fibers such as flax, hemp, bamboo, kenaf, silk, sisal, coir, and ramie are among the most commonly used organic fillers [15]. Biocomposites based on thermoplastic starch and cellulose fibers are very attractive materials. They are ecological and have good mechanical properties, which is why they are widely used in the packaging and automotive industries [16,17]. In research, flax fibers are widely used, primarily due to their relatively low density and low cost, acceptable specific properties, biodegradability, improved energy recovery, ease of separation, and growing environmental awareness. Other flax-fiber-reinforced polymers are used in various industries such as packaging, pharmaceutical, biotechnology, horticulture, or automotive. However, natural fibers in biocomposites have a few disadvantages, including poor compatibility between natural fibers and biodegradable matrices, and the high ability of natural fibers to absorb moisture, which is due to their cellulosic structure. As a result, modifications to natural fibers are being considered to change their surface properties and improve their adhesion to biodegradable matrices.

There are two main methods of fiber modification (chemical and physical). Chemical treatment by mercerization, silanes, maleic acid, or acetylation is used for the cleaning and/or modification of surface energy to enhance the interfacial adhesion with the polymer matrix [15,18]. The chemical surface modification leads to improved fiber dispersion and promotes proper interfacial bonding because it reduces voids and increases fiber distribution [19,20]. However, these treatments are time and cost-consuming processes that also impose an ecological risk. A sustainable alternative for chemical treatment offers a plasma or corona surface treatment and/or the use of ionizing radiation like electron beam (EB) [15,21,22,23,24,25].

As mentioned, the hydrophilic nature of fibers is a major problem for all natural fibers if used as reinforcement in polymers. The physical properties of each natural fiber are critical and include the fiber dimensions, defects, strength, and structure. A high aspect ratio (length/width) is very important in cellulose-based fiber composites as it indicates possible strength properties. Also, fiber dispersion is an important factor influencing the properties of fiber-reinforced polymers. Poorly dispersed fibers provide little and zero reinforcement in some regions, which negatively affects the strength of the composites [26].

Fiber strength can be an important factor in selecting a specific natural fiber for a specific application. Fiber dimensions, defects, strength, crystallinity, and structure should be taken into consideration. In our own research, a special configuration of screws was applied, intended to minimize the cutting of the fibers. This did not involve the elements returning and intensively mixing the polymer melt, but they were replaced by the main elements transporting the melt.

There is a growing interest in natural plant compounds that have antimicrobial properties, particularly in light of the need to sterilize various items such as seats, medical equipment, food packaging, and pharmaceuticals during the ongoing pandemic. Currently, gamma radiation and chemical disinfection methods are commonly used to sterilize these items, but they can be expensive, time-consuming, and may not be effective on all materials. Additionally, the chemicals used for disinfection can change the properties and durability of the materials and may have a negative impact on human health. Using natural plant compounds that exhibit biocidal activities can be a better option as they do not adversely affect the physical and chemical properties of the biocomposites. They may even improve the mechanical properties and biodegradation rate of the materials [27,28,29,30,31]. Therefore, it is justified to explore the use of natural plant compounds as a safer and more effective alternative for sterilization purposes. These compounds have been used for centuries in natural medicine and one can expect that, when used for the sterilization of biocomposites, they will not be harmful to the consumers’ health. Therefore, tannic acid was used to modify the fibers in the research.

Tannic acid (TA) is a natural polyphenol found in various plants, fruits, tea leaves, and red wine [28,32]. It contains one or more hydroxyl groups connected to an aromatic ring. TA has antioxidant properties and is effective against bacteria or fungi [33,34,35,36,37,38,39]. It can be used as a modifier, such as a cross-linking agent, and at the same time as an antibacterial agent, and can improve the hydrophobicity of biocomposites [29]. Hydrophobicity is important for materials exposed to microorganisms (e.g., packaging), as a high degree of surface hydrophobicity reduces the likelihood of colonization. This results in longer service life for finished products. Additionally, tannic acid can inhibit internal and external antioxidant processes. Due to its biocidal, antioxidant, and cross-linking properties, tannic acid is a promising modifier for polymer biocomposites, particularly in the packaging industry.

The scientific purpose of this paper was to develop novel biocomposites with biocidal properties on microorganisms and with enhanced mechanical strength, as well as with increased biodegradation rates. The main idea and the novelty of this work was to use cross-linking compounds and, at the same time, biocidal compounds—natural compounds of plant origin with biocidal properties.

## 2. Materials and Methods

### 2.1. Materials

Potato starch obtained from the Potato Industry Company (Trzemeszno, Poland) was used as the main component of the composites. Glycerine (PolAura, Zawroty, Poland); a molecular weight (MW) of 92.1 g/mol was used as a plasticizer. Then, 20 wt% of flax fibers (Ekotex, Namysłów, Poland), with a maximum length of 5 mm, were added to the matrix. Tannin acid (Sigma-Aldrich, Poznań, Poland) at a concentration of 20 wt% (aqueous solution) with MW = 1701.20 g/mol was used to modify the fibers. For the enzymatic degradation test, the enzyme *Proteinase K* from *Tritirachium album* (Blirt, Gdańsk, Poland), buffer 0.1 M Tris HCl, and sodium azide (NaN_3_) were used. For microbiological testing, *Staphylococcus aureus ATCC 6538P* and *Escherichia coli ATCC 8739* bacterial strains were used. For biocidal activity testing, Propidium iodide and CYTO Green (Invitrogen, Waltham, MA, USA) were used.

### 2.2. Sample Preparation

**Figure 1:**
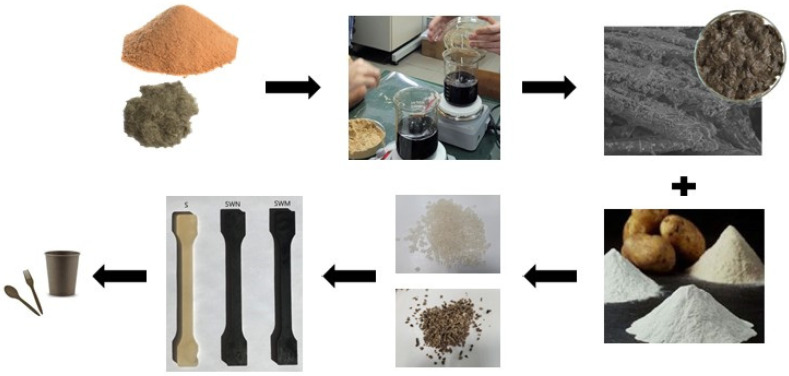
Stages of preparation of biocomposites.

The biocomposites were prepared through four phases: fiber treatment, preparation of potato starch and TPS, mixing all components, and extrusion, injection, or pressing. The individual stages are illustrated in Figure 1.

#### 2.2.1. Fiber Treatment

The flax fibers were dried for twelve hours at 60 °C before being modified in the tannic acid solution. To prepare a 20% (wt%) water solution of tannin acid, we used a magnetic stirrer (LLG-uniSTIRRER, Meckenheim Germany). We mixed distiller water and tannic acid at 1500 rpm for 15 min at 80 °C. After that, we lowered the temperature of the solution to 50 °C and mixed it for an hour to make the solution more homogeneous.

In the next stage, the dried flax fibers were coated with the TA solution. The fibers were soaked in the solution for 12 h at room temperature and then put on a sieve to remove excess solution for the next 12 h. Ultrasound was used for 12 h during the immersion process, with a power of 320 W and a frequency of 40 kHz. Finally, the fibers were dried for 24 h at 60 °C. According to the text, this method of fiber treatment was based on a patent [40].

#### 2.2.2. Preparation of TPS

Potato starch (75 wt%) was mixed with glycerine (25 wt%) conventionally (in a ziplock bag). The obtained mixture was placed on a laboratory sieve shaker with a vibration frequency of 50 Hz to break down the starch particles, better penetrate the plasticizer, and swell the starch. The TPS (thermoplastic starch) was produced using a co-rotating twin-screw extruder (20/40D) with granulation (Bühler, Braunschweig Germany). The extruder was operated at a temperature of 100 °C and a rotational speed of 95 rpm. To prepare dumbbell- and bar-shaped samples for mechanical testing, an Engel Victory 120 hydraulic injection molding machine was used. The barrel heating zones of the injection molding machine were set to different temperatures: 110, 120, 130, 135, and 35 °C of the mold.

#### 2.2.3. Mixing All Components

Potato starch (75 wt%), glycerine (25 wt%), and modified fibers (20 wt%) were blended and added to the extruder feeding zone. The temperatures of all profiles of the extruder were 100 °C and 95 °C of the head. After that, dumbbell- and bar- samples were prepared for mechanical testing using a hydraulic injection molding machine Engel Victory 120. The barrel heating zones of the injection molding machine were set to 130, 140, 155, 165, and 35 °C of the mold to prepare the samples.

The obtained biocomposite granulate was also pressed on the PHM-63 press. The frame was 30 by 30 cm. Table temperature was 160 °C. The ironing was achieved in stages: I—no pressure (heating and venting) for 3 min, II—pressure 30 bar for 1 min, III—70 bar for 1 min, and IV—120 bar for 1 min. Cooling for 10 min.

The method used for the preparation of TPS and biocomposites was based on the description provided in the patent application [41].

The samples are indicated as follows: TPS, TPS_FN, and TPS_FM, where TPS indicates thermoplastic starch (potato starch + glycerine), TPS_FN refers to a type of material that contains non-modified flax fibers, TPS_FM contains flax fibers that have been modified using tannic acid, FN stands for non-modified flax fibers, and FM stands for modified flax fibers.

### 2.3. Methods

The Q 800 DMA analyzer from TA Instruments, New Castle, DE, USA was used to perform Dynamic Mechanical Analysis (DMA). Bar-shaped injection-molded samples were examined in a dual cantilever mode at a constant frequency of 1 Hz and a controlled amplitude of 15 μm. The analysis was carried out over a temperature range of 25 to 160 °C.

Tensile strength and Young’s modulus were determined using a tensile testing machine, type Instron 3367 (Instron, Norwood, MA, USA).

The thermogravimetric (TG) measurements were performed in a nitrogen atmosphere, using a Q500 thermogravimetric analyzer (TA Instruments, New Castle, DE, USA). The mass of individual samples was about 19 mg. The TGA measurements were carried out in a temperature range from 20 to 800 °C and with a heating rate of 10 °C/min.

Differential scanning calorimetry (DSC) measurements were conducted using a TA Instruments (New Castle, DE, USA) Q200 differential scanning calorimeter in a nitrogen atmosphere. Individual sample masses were about 8 mg, and the measurement temperature range was from 0 to 210 °C, at a heating rate of 10 °C/min. The test was carried out using the heating–cooling–heating scanning. To eliminate the thermal history of the samples, the measurement results were analyzed based on the data from the second heating.

To determine the contact angles of the specimens involved, a goniometer (Krüss GmbH, Hamburg, Germany) equipped with an automated dosing drops system was used. Water (as the test liquid) at a volume of 7 μL was placed on the specimen surface at an inflow rate of 50 μL/min. The contact angle of each sample was measured six times for accuracy.

Images of the morphology of granules of native potato starch and TPS were recorded by using an optical microscope VHX7000 (KEYENCE, Mechelen, Belgium). The geometric structure of the tested surface was analyzed using an optical microscope and scanning electron microscope (SEM) Phenom XL from ThermoFisher Scientific (Eindhoven, The Netherlands), which captured images of the changes. Additionally, a 2 nm layer of gold was applied to the samples before the measurement.

To create thin film samples (approximately 0.5 mm thick) for microbiological testing, a vulcanization press (AW03M, Argenta, Brzeziny, Poland) was used. About 1 g of composite granules was introduced into the press (at 180 °C) and pressed for 10 s at a pressure of 0.7 MPa. Once the pressure process was complete, the samples were removed from the press and left to cool.

The biocomposites that were prepared went through evaluation to determine their bactericidal properties. This evaluation was performed following the standard ISO 22196:2011 Measurement of antibacterial activity on plastics and other non-porous surfaces, which measures antibacterial activity on plastics and other non-porous surfaces. This study used reference bacterial strains, namely *Staphylococcus aureus* (ATCC 6538P) and *Escherichia coli* (ATCC 8739), and was carried out in triplicate. TPS and TPS_FN films were used as control samples. The samples were produced in the form of thin films using a vulcanization press, as described above.

During the research, the bacterial strains under investigation were placed on both control films and samples. A specific number of cells were left for a set period, with no time for validation of recovery efficiency and 24 h for testing. After this time, bacterial cells were retrieved from the surface and then suspended in a solution containing a neutralizer (soybean casein digest broth with lecithin). The number of viable and growing cells was then determined by inoculating them on Plate Count Agar in triplicate, which was then incubated for 24 h at 37 °C. The reduction (R) in the number of living and viable cells of tested bacteria was calculated using the following Equation (1):

R = (U_t_ − U_0_) − (W − U_0_) (1)

where U_0_ means the logarithm of the number of viable bacteria obtained from the control samples just after inoculation and U_t_ means the logarithm of the number of viable bacteria obtained from the control samples after 24 h. It serves as a measure of survival in time. Lastly, W represents the mean of the logarithm of the number of viable bacteria obtained from the samples after 24 h.

According to standard ISO 22196, a bactericidal effect of a composite material can be achieved if the reduction in the number of cells capable of growth is by two orders of magnitude or more (R ≥ 2).

The biodegradation process was conducted under industrial composting conditions at the Segregation and Composting Plant in Zabrze, Poland. Samples of the material were placed on racks in specially designed baskets made of stainless steel with dimensions of 27 × 70 × 21 cm (width × length × height). The research was carried out on a separate part of the static compost pile consisting of leaves (about 40%), wood chips (about 30%), and grass (about 30%). The dimensions of the prisms were 30 × 33 m with a height of about 4 m. The organic waste mixed in the mixer was laid on a naturally aerated substrate made of concrete slabs equipped with holes, allowing for aeration of the pile without the need to overturn the biomass. The sample baskets were buried at a depth of 1 m. The biodegradation process was carried out for 3 weeks with an average temperature of about 65 °C. After appropriate incubation times of 7, 14, or 21 days, the baskets were removed from the heap. Due to the very fast biodegradation process and the decomposition of samples, after 3 weeks in baskets, it was not possible to collect samples.

## 3. Results and Discussion

**Figure 2:**
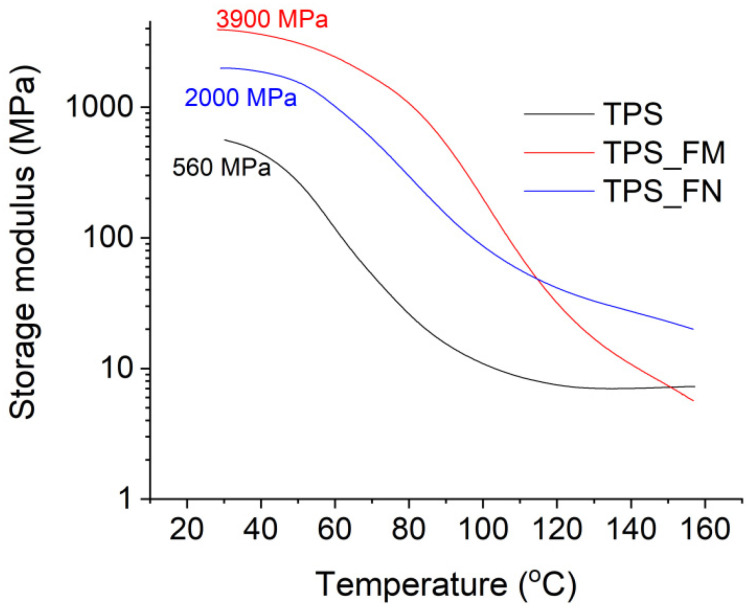
Storage module of TPS, TPS_FN, and TPS_FM samples as a function of temperature.

**Figure 3:**
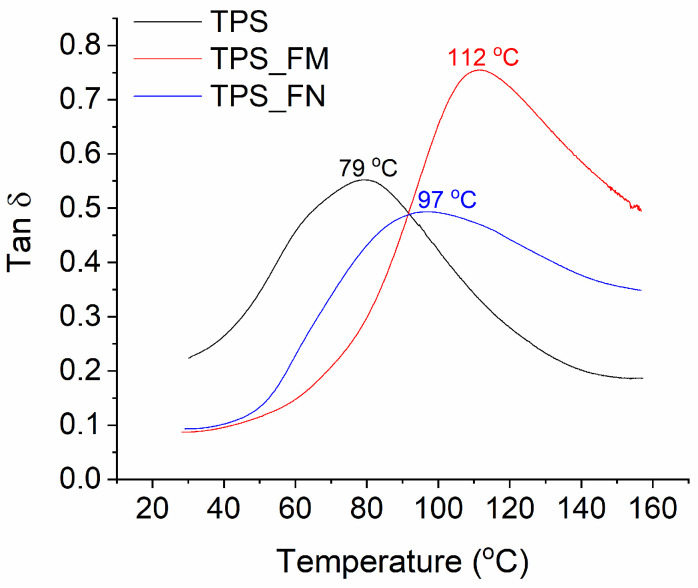
Damping coefficient (tanδ) of S, SWN, and SWM samples as a function of temperature.

The results of the DMA analysis are presented in Figure 2 and Figure 3. Figure 2 presents the changes in the storage module as a function of the temperature for samples TPS, TPS_FN, and TPS_FM. The storage modulus of sample TPS was approximately 560 MPa at room temperature. After the introduction of flax fibers (TPS_FN sample), an increase in this value by over 250% was observed (up to about 2000 MPa). The increase in stiffness was obviously due to the reinforcement of fibers whose modulus is many times greater than that of the starch plasticized with glycerine. Modification of the fibers with tannic acid contributed to a further increase in the storage modulus by up to about 3900 MPa (TPS_FM sample) at room temperature conditions. This may prove the improvement of the interfacial adhesion of fibers modified with tannic acid, to which the stresses from the polymer matrix were effectively transferred. This is closely related to the presence of large amounts of catechol and galloyl groups within molecular TA. Waite et al. [42] demonstrated that catechol groups played a key role in the adhesion process. Due to the catechol structure, TA shows strong adhesion to various object surfaces such as fibers, plastic, and metal [43,44].

The damping factor (tanδ) can be a good indicator of the mechanical energy being absorbed by macromolecular segments in their translational movements. As can be seen from Figure 3, the glass transition temperature (T_g_) of the starch sample was about 79 °C, while after adding flax fibers, its value increased to about 97 °C. The increase in T_g_ temperature probably resulted from the limited movement of starch macromolecules at the interface of the starch/flax phases. The modification of fibers with tannic acid caused a further increase in T_g_ of up to about 112 °C, which can also confirm the greater interfacial adhesion of modified fibers than unmodified ones. However, in the case of modified fibers (TPS_FM sample), an increase in T_g_ was accompanied by a significant increase in the damping coefficient. This may prove that some of the tannic molecules migrated to the starch matrix and facilitated the mutual translational movement of starch molecules mainly during phase transition (from glassy to elastic).

As presented in Table 1, tensile tests revealed that after the addition of flax fibers, Young’s modulus increased from about 883 MPa (sample TPS) to about 1594 MPa (sample TPS_FN), while tensile strength remained statistically unchanged. However, a significant decrease in the strain at maximum load was observed, which was the same as the strain at break. The reason for this could be disturbances in the continuity of the polymer matrix structure at the interface with the fibers (internal microcarbs). A much better load transfer, especially in the elastic range, can be seen in the case of the sample containing modified fibers, whose modulus of elasticity increased up to 3378 MPa. In terms of non-elastic (plastic) deformations, a decrease in the strain at break/maximum load was similar to the sample with unmodified fibers (sample TPS_FN).

The thermal stability of biocomposites is a crucial factor in their production process. Natural fibers have low thermal stability and are therefore only suitable as fillers for materials that are processed at temperatures below 200 °C [45]. In the developed method of producing biocomposites (patent application P.444007), the processing temperatures do not exceed 100 °C (in the case of extrusion), 135 °C degrees (injection), or 165 °C degrees (press); therefore, there is no threat of degradation of plant fibers.

When using natural fibers as a reinforcement in polymer-based materials, it is important to remember that the temperature during extrusion and injection molding plays a vital role in determining the mechanical properties of the composites. High temperatures can cause the natural fibers to degrade, leading to undesirable effects such as discoloration or a decrease in mechanical strength [21,46].

**Figure 4:**
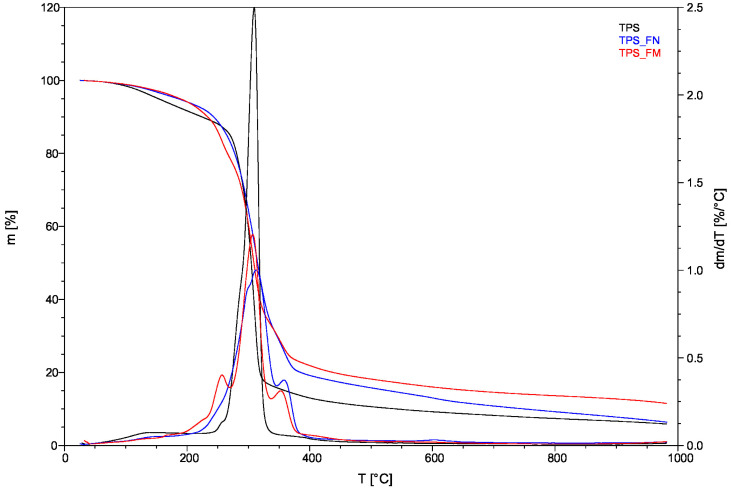
The TG curve and DTG curve for the TPS, TPS_FN, and TPS_FM.

Figure 4 illustrates the thermal decompositions of the biocomposites. Table 2 summarizes the temperatures of decomposition (Td), as well as the temperatures for mass losses of 5%, 25%, 50%, and 95% (T_5%_, T_25%_, T_50%_, and T_95%_, respectively), which were derived from TG and TG/DT curves.

The TPS thermogram displayed a gradual decrease in weight as the temperature increased up to approximately 130 °C. This is largely due to the evaporation of moisture. Afterward, the TPS curve continued to decline due to glycerol decomposition at 256.43 °C, reaching the point of starch decomposition at around 309 °C.

TPS_FN and TPS_FM curves also show a slight weight loss related to moisture loss and the evaporation of small particles such as water (present in fibers) or glycerine.

Thermal decomposition occurs between 150 °C and 400 °C. DTG curves indicate that the peak temperatures of the maximum decomposition rates (T_d_/T_dt_) of TPS_FN and TPS_FM were 312.68 °C and 307.13 °C, respectively. However, the thermal decompositions of the individual compositions contained in the samples overlap. The weight loss corresponds to the degradation of tannic acid, flax fiber, starch, and glycerol.

Comparing the TPS_FM and TPS_FN curves, it can be seen that the TPS_FM has a few thermal zones (three more than TPS_FN) which is consistent with the results [47]. This is associated with the process of tannic acid degradation which occurs in three stages. The first stage involves the dehydration of tannic acid at temperatures below 150 °C. The second stage involves the decarboxylation of the galloyl moieties present in the outer layer between the temperature range of 240 °C and 340 °C. The third and final stage involves the degradation of galloyl moieties at the inner layer and the central glucose group at temperatures above 340 °C.

In the first TPS_FM zone (to about 150 °C), the oxidation stage, elimination of volatile fractions occurs, as well as the dehydration of TA. In the second zone (to about 200 °C), the first depolymerization of TA is carried out, with a pronounced peak at 167.86 °C on the DTG curve. Between 200 °C and 270 °C is presented a second depolymerization of the material. The third degradation of TA takes place after 272 °C to 352 °C.

The main peaks visible on the DTG curve at about 257 °C and 350 °C were wide, which may indicate the overlapping decomposition between individual components contained in biocomposites, such as hemicelluloses, cellulose, lignin, TA groups, starch, and glycerol. Decomposition reactions can occur simultaneously. The peak (about 257 °C) suggests the overlapping decomposition of hemicellulose and pectins (from fibers) or glycerol (from TPS), and also decarboxylation of the galloyl moieties in the outer layer (from TA). Whereas the peak of about 350 °C suggests the overlapping of cellulose decomposition (from fibers) and the degradation of the glucose and galloyl groups in the outer layer from TA.

The TPS_FN sample undergoes rapid weight change due to fiber decomposition during the pyrolysis process between 230 °C and 400 °C.

**Figure 5:**
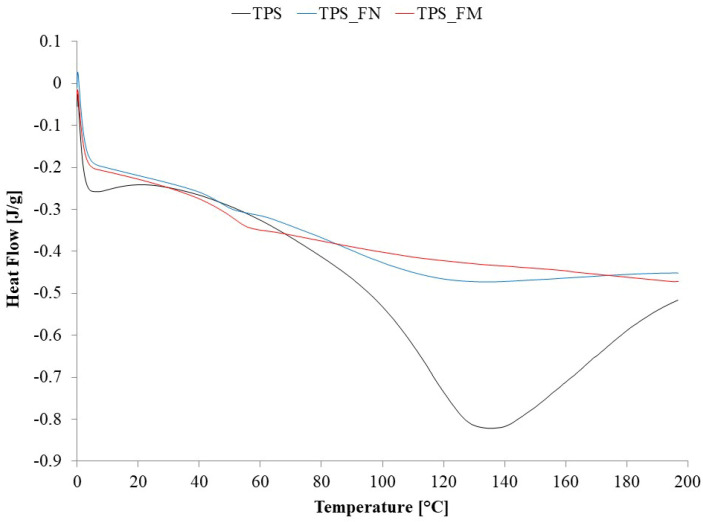
The temperature dependence on the heat flow (DSC solid curve—second heating cycle) for the TPS, TPS_FN, and TPS_FM.

The results of the DSC analysis are presented in Figure 5.

In the sample TPS curve, there is one broad endothermic peak with a T_m_ of 131.2 °C and a ΔH_m_ of 164.8 J/g. According to [48,49,50], the peak is related to the melting of starch crystals in thermoplastic starch. After the introduction of flax fibers into the TPS, the observed endothermic peak significantly decreases with the simultaneous appearance of a visible step change at 45.6 °C, which is due to the glass transition of the amorphous phase of the TPS matrix. Thus, it can be seen that the introduction of the fibers limited the possibility of the movement of the TPS macromolecules, which resulted in a reduction in the crystalline phase in the material and, as a result, an increase in the amorphous phase content. When analyzing the TPS_FM sample curve, it seems that modifying the fibers with tannic acid increased their adhesion to the polymer phase of composites, as the previously observed endothermic peak disappears completely, and the transformation associated with the glass transition occurs at a much higher temperature, i.e., 52.4 °C. The observed increase in the glass transition temperature indicates an even greater limitation of the movement of TPS macromolecules as a result of fiber modification.

The results for the water contact angle (Θ_w_) of the tested samples are presented in Table 3. The comparison between the Θ_w_ values of TPS (32.8) and TPS_FM (81.7) indicates that the presence of tannic acid, a natural biocidal factor, has a positive impact on the hydrophobicity of TPS_FM. TA causes an increase of nearly 49° in the Θ_w_ of TPS_FM as compared with TPS. Natural fibers’ surface properties can be changed by tannic acid. It can form a barrier on the fiber’s surface, making it less porous and protecting it from moisture. The decreased water absorption capacity of the fiber is one effect of the surface changes along with the cross-linking phenomena [51].

**Figure 6:**
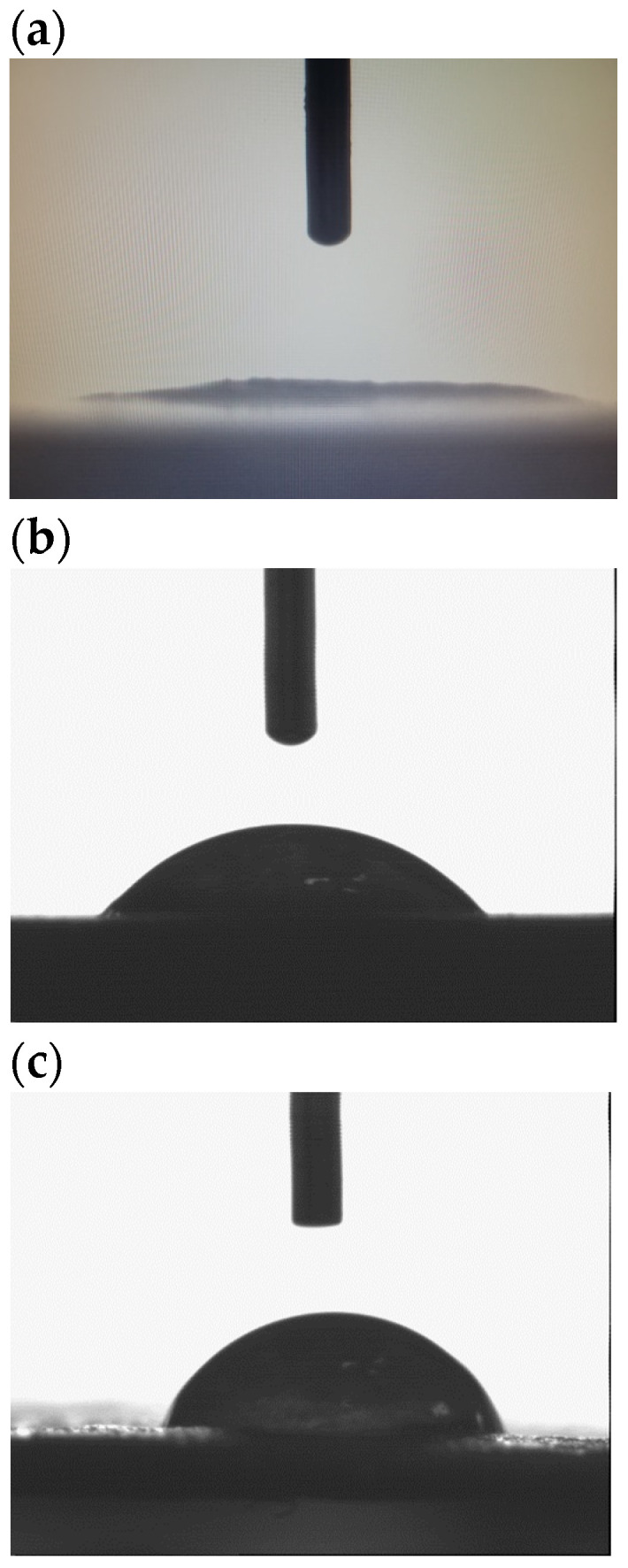
The water drop placed on the sample: (**a**) TPS, (**b**) TPS_FN, (**c**) TPS_FM.

Figure 6 shows the shape of a drop of water on the tested samples. The drop shape on the surface of the TPS_FM sample is more spherical than the drop shape on the surfaces of the TPS and TPS_FN samples. In the case of the TPS sample, water was absorbed into the sample very quickly.

The incorporation of TA as a modifier of flax fibers has a positive impact on the hydrophobicity of the biocomposites produced. This is particularly important for food packaging materials, where it is necessary to prevent any liquid leakage, such as in single-use cups. Furthermore, increased hydrophobicity is also crucial for materials that are exposed to pathogens, as it reduces the likelihood of microbial colonization. As a result, the shelf life of the final products is automatically extended.

**Figure 7:**
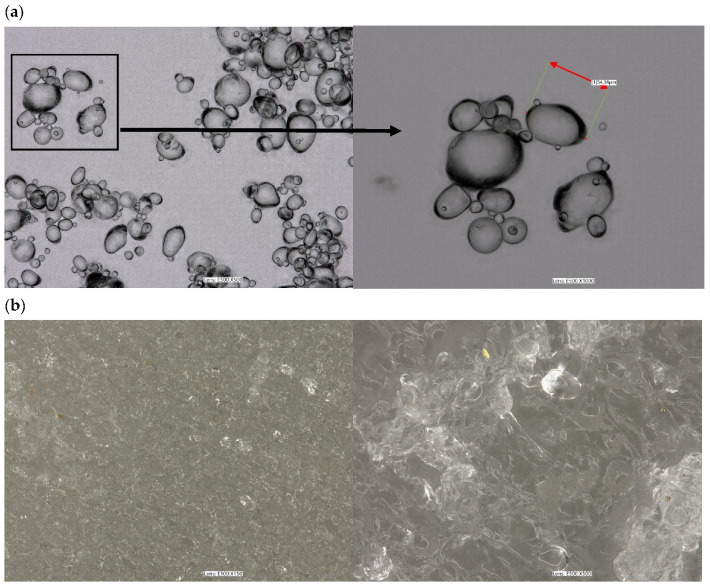
Optical microscope images: (**a**) native potato starch, (**b**) TPS.

Figure 7 shows the optical microscope images that showcase the granule morphology of native potato starch and TPS. The images reveal the impact of plasticizing and extrusion on the granule morphology of native starches. In Figure 7a, it can be observed that the native starch granules were intact and had distinct oval to elliptical shapes. Whereas plasticizing the starch with glycerol and an extrusion process resulted in the partial destruction of the starch structure, granule fragmentation, and the smoothing of its surface. However, on the surface, TPS can still be seen in the presence of some grains (Figure 7b). Similar results were observed in works [1,4,52].

**Figure 8:**
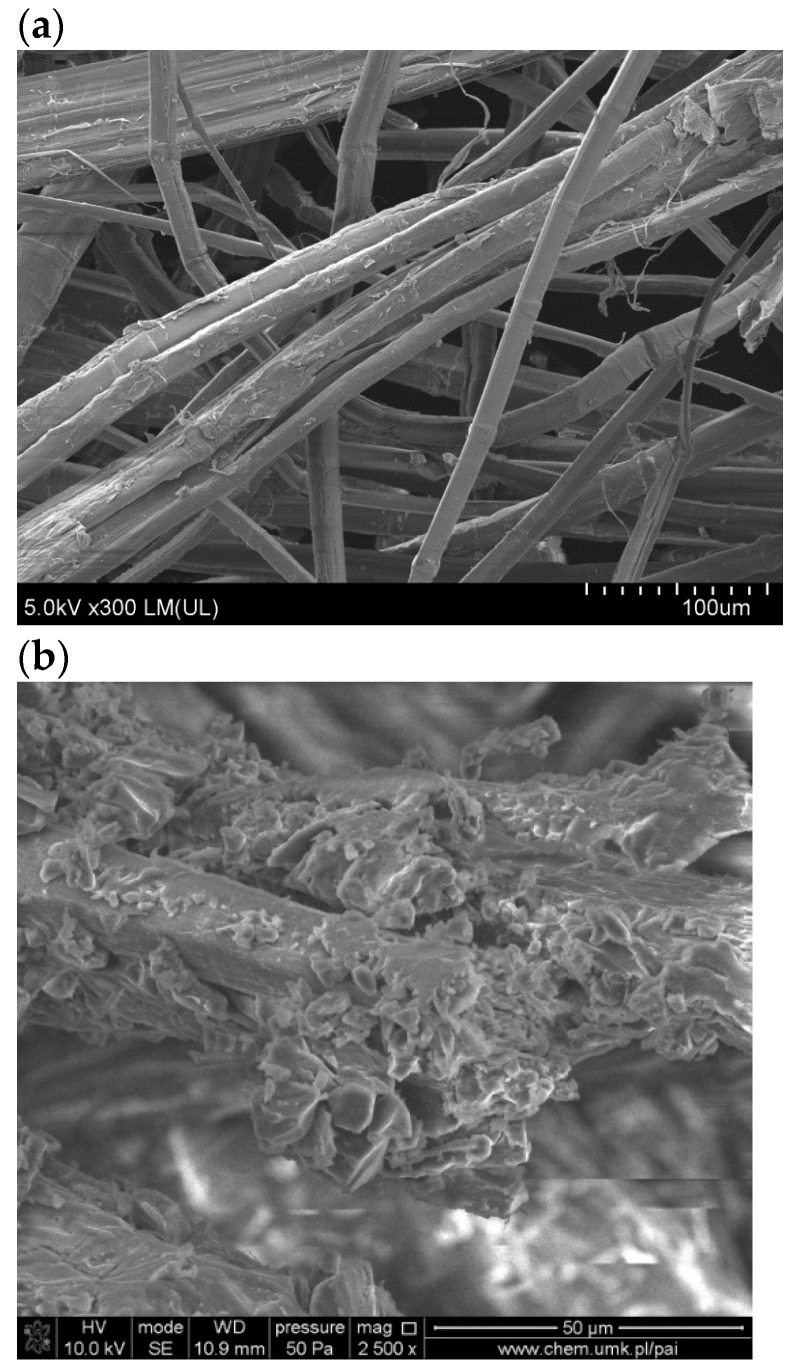
SEM images of the surface of fiber: (**a**) unmodified, (**b**) modified TA.

**Figure 9:**
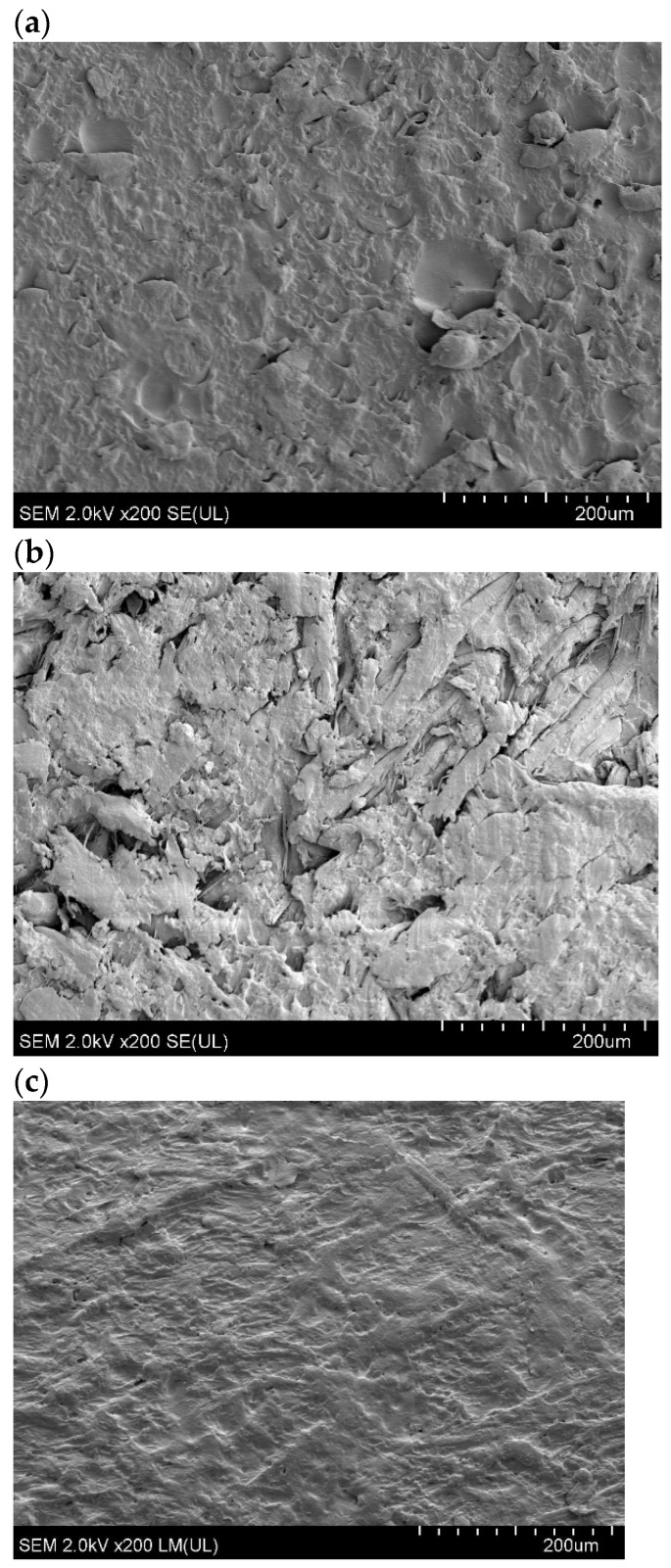
SEM cross-section images: (**a**) TPS, (**b**) TPS_FN, and (**c**) TPS_FM.

Figure 8 presents the SEM images of the surface of FN and FM. As seen in Figure 8b, flax fibers are covered with tannic acid. Figure 9 shows the SEM cross-sectional images of TPS, TPS_FN, and TPS_FM composites.

Figure 9a shows that the cross-section of pure TPS is mostly flat, with visible unplasticized natural starch granules. In contrast, the biocomposite in Figure 9b has fibrous and granular structures. The TPS_FN in the biocomposite appears as clumps, indicating poor dispersion of FN in the blend. The empty spaces observed in the SEM pictures are due to weak interfacial interactions between the blend components [53]. However, in the composite with flax fibers containing tannins, the particles of incompletely plasticized starch were reduced. The samples were flatter and smoother than TPS or TPS_FN, and no starch granules were visible. These observations suggest better interfacial interactions of the blend. Holes, cracks, and protruding elongated fibers are visible in the TPS_FN biocomposite, indicating poor interfacial adhesion with the TPS matrix (Figure 9b).

After modifying the fibers with tannin, the surface of the biocomposite appeared more homogenous and had fewer holes and protruding fibers, as seen in Figure 9c. This suggests that the fibers were broken rather than being pulled out, which could be due to the improved adhesion at the interface. This effect may be attributed to the active role of tannic acid.

To quantitatively evaluate interfacial adhesion, micromechanical tests such as single fiber pull-out or fragmentation experiments should be performed [54]. This will be the focus of future studies.

The reductions (R) in *Escherichia coli* and *Staphylococcus aureus* bacterial strains for the tested samples are presented in Table 4.

According to the ISO 22196:2011 standard, a biocidal agent can be considered effective if the R ≥ 2. This study revealed that TPS_FM was able to effectively combat the bacterial strains of Escherichia coli and Staphylococcus aureus. These results fulfill the requirements of the ISO 22196:2011 standard, thus confirming that tannic acid can be considered as a biocidal factor.

**Figure 10:**
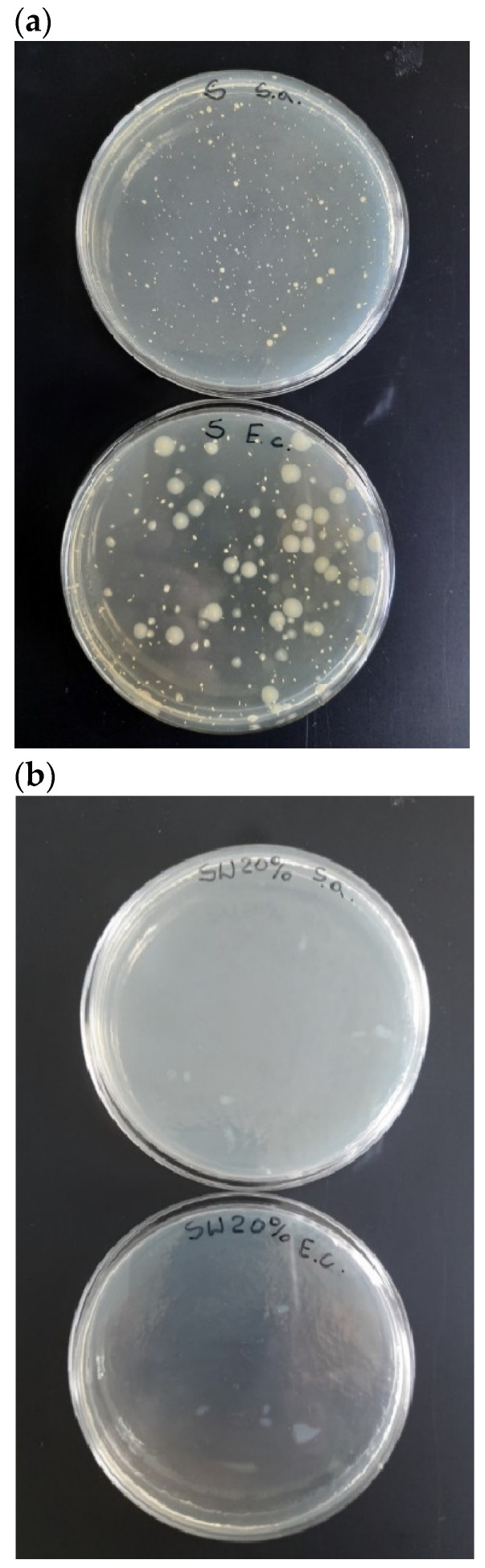
The Petri dish images: (**a**) inoculation on agar, (**b**) after incubation for 24 h.

The biocidal effect of tannic acid is confirmed by the images of the Petri dishes for TPS and TPS_FM samples (Figure 10). It clearly shows that for the TPS_FM sample, there was an inhibition zone for *E. coli* and *S. aureus* (no shown bacteria).

Determination of the biocidal activity of the prepared biocomposites is vital from the point of view of predicting which microorganisms a given sample would be biocidally active against and, thus, what potential applications of the studied material could be.

**Figure 11:**
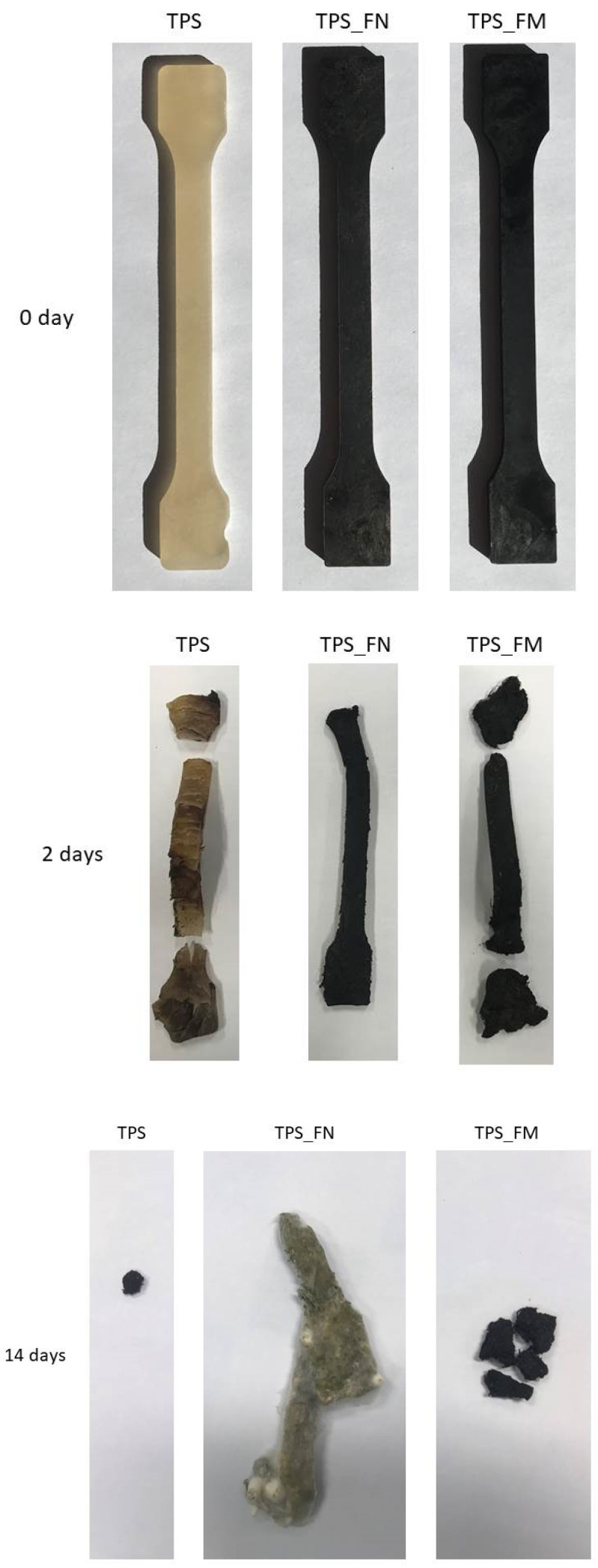
Pictures of the biodegradability process of TPS, TPS_FN, and TPS_FM in industrial composting by 0, 2, and 14 days.

Figure 11 displays images of TPS, TPS_FN, and TPS_FM biodegradability processes in industrial composting. The reference samples have a relatively smooth surface without visible cracks.

After just two days of composting, the samples underwent significant changes—they began to crack and fragment. This was due to the hydrophilic nature of starch, which caused the samples to absorb a large amount of water. As a result, they were invaded by microorganisms present in the composting environment.

Next, after 14 days of composting, the pure TPS and TPS_FM samples were further fragmented into several pieces and almost completely disintegrated. The appearance of the TPS_FN sample (shape and color) changed significantly but did not fragment into small pieces in this case. Most likely, the color change was caused by the decomposition and degradation of the fibers in the biocomposite. The fibers present in the sample support the growth of fungi by acting as capillaries that help in the transportation of nutrients from the external surfaces or vulnerable regions, leading to extensive colonization by microbes. The observed change in the shape of the sample was due to the high temperature in the composter, which caused the relaxation of stress after processing.

After 21 days of composting, all samples were completely degraded and no pieces of the samples were recovered. The results indicate that tannic acid does not negatively affect the rate of biodegradation, which is different from the reported work [55] where the presence of high amounts of tannin reduced the biodegradability rate in the soil due to tannins’ broad antimicrobial properties. We believe that the possible negative impact of tannin on degradation processes is not due to antimicrobial properties as reported [55] but only due to the cross-linking properties of TA and increased hydrophobicity samples. The higher the hydrophobicity, the lower the ability to migrate water and microorganisms, such as fungi and bacteria, inside the material. Our research, despite the increased hydrophobicity of the biocomposite, showed an increased rate of biodegradation in the biocomposite with TA compared with the biocomposite without TA (see Figure 10, 14 days). Ultimately, we saw no negative effect of TA on the rate of biodegradation (after 21 days no piece of the sample was recovered). Similar conclusions to ours were drawn in Cheng’s work [56].

## 4. Conclusions

Biodegradable polymers are an innovative material in the field of packaging. Therefore, efforts were made to develop novel biocomposites of biocidal properties in microorganisms and of enhanced mechanical strength and hydrophobicity, as well as with increased biodegradation rates. Biodegradable polymers that contain starch and natural fibers are expected to experience a substantial increase in their usage. This is because the biocomposites produced from these materials are renewable, cheap, and readily available. For packaging materials, the most crucial aspect is the hydrophobic nature of the surface and their biocidal properties.

A natural plant compound has been used to modify flax fiber and create new biocomposites with reduced hydrophilicity. This is desirable in industries such as packaging, disposable products, horticulture, medicine, and beauty, where sterility and microbiological hygiene are important. The hydrophobic surface of these materials makes them less susceptible to colonization by bacteria and inhibits the growth of pathogens.

The results showed that tannic acid-treated flax fiber is effective in fighting Escherichia coli and *Staphylococcus aureus* bacteria. The standard ISO 22196:2011 considers tannic acid to be a biocidal factor and the produced biocomposite to be a biocidal material. Determination of the biocidal activity is very important and vital from the viewpoint of predicting which microorganisms a given sample would be biocidally active against and, thus, what potential applications of the studied material could be. Additionally, due to its non-toxicity, the biocomposite can be used in food-contact applications, such as catering and packaging industries.

## Figures and Tables

**Table 1:** Young’s modulus, tensile strength, and strain at maximum load of sample TPS, TPS_FN, and TPS_FM.

Sample	E [MPa]	σ_M_ [MPa]	ε_M_ [%]
TPS	883 ± 35	14 ± 3	2.3 ± 0.8
TPS_FN	1594 ± 130	15 ± 2	1.2 ± 0.2
TPS_FM	3378 ± 170	31 ± 5	1.4 ± 0.3

**Table 2:** Temperatures of decomposition (T_d_) and temperatures (T_5%_, T_25%_, T_50%_) correspond to mass losses of 5%, 10%, and 50%, derived from TG and DTG curves.

Sample	T_d_ [°C]	T_d/dt_ [°C]	T_5%_ [°C]	T_25%_ [°C]	T_50%_ [°C]
TPS	291.11	309.15	152.93	286.86	304.68
TPS_FN	274.51	312.68	184.90	286.56	314.97
TPS_FM	277.72	307.13	188.13	280.12	308.87

**Table 3:** The Θ_w_ values of the tested samples.

Sample	Θ_w_ [°]
TPS	32.8
TPS_FN	61.1
TPS_FM	81.7

**Table 4:** The reductions (R) in bacterial strains.

Tested Bacteria *	Samples					
	S		SWN		SWM	
	W	R	W	R	W	R
*E. coli*	4.9 × 10^6^	0	0.5 × 10^6^	0	0.5 × 10^3^	3
*S. aureus*	4.5 × 10^6^	0	8 × 10^6^	0	0.5 × 10^3^	3

* The numbers of cells of the tested strains in the prepared suspension for testing: *E. coli* 9.75 × 10^6^ jtk/mL; *S. aureus* 15 × 10^6^ jtk/mL. The time of the contact of bacteria with the tested foil was 24 h.

## Data Availability

Data are contained within the article.
